# Efficiency analysis for quantitative MRI of T1 and T2 relaxometry methods

**DOI:** 10.1088/1361-6560/ac101f

**Published:** 2021-07-26

**Authors:** David Leitão, Rui Pedro A. G. Teixeira, Anthony Price, Alena Uus, Joseph V. Hajnal, Shaihan J. Malik

**Affiliations:** 1 School of Biomedical Engineering and Imaging Sciences, King’s College London, London, United Kingdom; 2 Centre for the Developing Brain, King’s College London, London, United Kingdom; 3 Communication Address: Perinatal Imaging and Health 1st Floor South Wing, St Thomas’ Hospital London SE1 7EHUK, United Kingdom

**Keywords:** efficiency, quantitative MRI (qMRI), T1 mapping, T2 mapping, steady-state, fingerprinting (MRF)

## Abstract

This study presents a comparison of quantitative MRI methods based on an efficiency metric that quantifies their intrinsic ability to extract information about tissue parameters. Under a regime of unbiased parameter estimates, an intrinsic efficiency metric $\eta $ was derived for fully-sampled experiments which can be used to both optimize and compare sequences. Here we optimize and compare several steady-state and transient gradient-echo based qMRI methods, such as magnetic resonance fingerprinting (MRF), for joint ${T}_{1}$ and ${T}_{2}$ mapping. The impact of undersampling was also evaluated, assuming incoherent aliasing that is treated as noise by parameter estimation. *In vivo* validation of the efficiency metric was also performed. Transient methods such as MRF can be up to 3.5 times more efficient than steady-state methods, when spatial undersampling is ignored. If incoherent aliasing is treated as noise during least-squares parameter estimation, the efficiency is reduced in proportion to the SNR of the data, with reduction factors of 5 often seen for practical SNR levels. *In vivo* validation showed a very good agreement between the theoretical and experimentally predicted efficiency. This work presents and validates an efficiency metric to optimize and compare the performance of qMRI methods. Transient methods were found to be intrinsically more efficient than steady-state methods, however the effect of spatial undersampling can significantly erode this advantage.

## Introduction

Over time many methods have been developed that aim to estimate ${T}_{1}$ and ${T}_{2}$ as effectively as possible, from classical inversion recovery and spin-echo sequences, to steady-state sequences (Deoni *et al* 2003, Welsch *et al* 2009, Heule *et al* 2014, Teixeira *et al* 2017, Shcherbakova *et al* 2018) and more recently MR fingerprinting (MRF) (Ma *et al* 2013). Selecting which method to favor for any given scenario can be challenging since the achieved precision (statistical uncertainty) and accuracy (proximity to true value), and the way these change with acquisition time are generally complex functions of the pulse sequence settings, tissue properties, specific details of the hardware used and the type of image reconstruction.

Nevertheless, comparisons have been made using metrics that strive to evaluate the intrinsic merits of each method (Crawley and Henkelman 1988, Jones *et al* 1996, Deoni Rutt and Peters 2003, Ma *et al* 2013, Assländer *et al* 2019a, Assländer 2020), accounting for the differences external to the methods themselves. Fundamentally, these differences are the amount of data and the SNR of the experiment. The amount of data has been normalized using either the total number of measurements (Jones *et al* 1996) or the total acquisition time (Edelstein *et al* 1983, O’Donnell *et al* 1986, Crawley and Henkelman 1988, Deoni *et al* 2003, Ma *et al* 2013, van Valenberg *et al* 2017, Assländer 2020). The SNR of the experiment has been normalized using the voxel volume (Deoni *et al* 2003), a combination of the thermal noise level with the proton density (Edelstein *et al* 1983, O’Donnell *et al* 1986, Jones *et al* 1996, van Valenberg *et al* 2017, Assländer *et al* 2019a, Assländer 2020) or the signal dynamic range of each method (Crawley and Henkelman 1988). Some of these studies (Ma *et al* 2013, Assländer *et al* 2019a, Assländer 2020) showed that balanced MRF (using a balanced readout) outperforms driven equilibrium (DE) single pulse observation of ${T}_{1}$/${T}_{2}$ (DESPOT) for both ${T}_{1}$ and ${T}_{2}$ estimation, but it is as yet unclear how other commonly used methods, like spoiled MRF (Jiang *et al* 2015) (using a gradient spoiled readout) or double echo steady-state (DESS) (Welsch *et al* 2009), compare. Furthermore, methods based entirely on gradient spoiled readouts are popular for their insensitivity to off-resonance at a cost of an SNR penalty—it is unclear whether this trade-off benefits ${T}_{1}$ and ${T}_{2}$ estimation.

In this work we focus on precision and propose a general efficiency metric that integrates the concepts from figures of merit used so far, quantifying the encoded information from a spin dynamics perspective, but not how it is decoded by the reconstruction. The resulting metric is then used to optimize and comprehensively compare a range of well-established methods that simultaneously estimate ${T}_{1}$ and ${T}_{2}.$ Finally, we discuss the utility of the efficiency metric and other important considerations when comparing qMRI methods.

## Theory

We consider that each voxel contains a single pool of spins characterized by unique values of ${T}_{1}$ and ${T}_{2};$ that the signal models for the different qMRI methods are accurate, subject to additive Gaussian noise; and that parameter estimation results in an unbiased estimate of the parameters of interest $\theta $ (i.e. ${T}_{1}$ and ${T}_{2}$ but potentially other parameters). Therefore, the error in the estimates is defined by the precision that is characterized by the standard deviation ${\sigma }_{\theta }$ of the estimated parameter $\theta .$ Analogous to the definition of SNR, the precision can also be represented by the parameter-to-noise-ratio ($\theta NR$):

\begin{eqnarray*}\begin{array}{c}\theta NR=\displaystyle \frac{\theta }{{\sigma }_{\theta }}.\end{array}\end{eqnarray*}

Although the $\theta NR$ directly relates to the SNR, it also depends on how much information about the parameter being measured is encoded in the data. Further, the SNR can be broken down to consist of an intrinsic SNR (relating to the receiver system, field strength, resolution etc) and the amount of data acquired. These dependencies have been highlighted in previous works (Edelstein *et al* 1983, O’Donnell *et al* 1986, Crawley and Henkelman 1988, Jones *et al* 1996) and can be expressed in the following equation that serves to define efficiency with which a parameter $\theta $ is estimated, $\eta (\theta ):$

\begin{eqnarray*}\begin{array}{c}\theta NR=\eta \left(\theta \right)\cdot SN{R}_{max}\cdot \sqrt{{T}_{acq}}.\end{array}\end{eqnarray*}

Here, $SN{R}_{max}\equiv {M}_{0}/{\sigma }_{0}$ represents the maximum SNR of any one measurement. ${M}_{0}$ is defined as the maximum signal from a voxel that would be measured by applying a ${90}^{\circ }$ pulse with the magnetization in thermal equilibrium; this is a characteristic of the system (field strength and coil) and acquisition geometrical parameters (resolution and field of view); ${\sigma }_{0}$ is the receiver noise standard deviation (i.e. what would be measured during one *k*-space data readout scaled to account for differences in scaling between *k*-space and image domain) and is characteristic of the *k*-space readout and its bandwidth; ${T}_{acq}$ is the total acquisition duration for all data required to estimate the parameter $\theta .$ Rewriting equation (2) with $\eta (\theta )$ as its subject gives:

\begin{eqnarray*}\begin{array}{c}\eta \left(\theta \right)=\displaystyle \frac{\theta NR}{SN{R}_{max}}\displaystyle \frac{1}{\sqrt{{T}_{acq}}}=\displaystyle \frac{\theta }{{\sigma }_{\theta }}\displaystyle \frac{{\sigma }_{0}}{{M}_{0}}\displaystyle \frac{1}{\sqrt{{T}_{acq}}}\leqslant \displaystyle \frac{\theta }{{\sigma }_{\theta }^{CRLB}}\displaystyle \frac{{\sigma }_{0}}{{M}_{0}}\displaystyle \frac{1}{\sqrt{{T}_{acq}}}.\end{array}\end{eqnarray*}

An upper bound on the efficiency can be obtained without need for physical measurement by calculating the Cramér–Rao lower bound (CRLB) for ${\sigma }_{\theta }\,\left({\sigma }_{\theta }^{CRLB}\right)$ (Sengupta and Kay 1995), resulting in $\eta \left(\theta \right)\leqslant {\eta }^{CRLB}(\theta ).$ The ability to achieve this bound depends on the full parameter reconstruction pipeline, which we assume to extract all encoded information so that equality holds.

**Figure 1.**
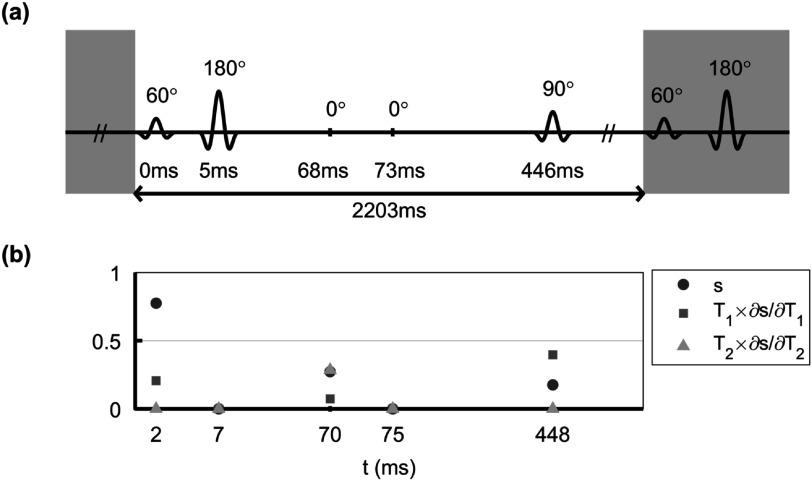
Optimized fingerprint with just 5 pulses that are applied cyclically, with a spoiling gradient preceding each pulse. Schematic representation of (a) the optimized flip angles and intervals between the pulses and (b) the signal $s$ and its derivatives w.r.t. ${T}_{1}$ and ${T}_{2}$ at echo time (2 ms). Note that the regions in gray are already repetitions of the main block of 5 pulses. Because the efficiency measure incorporates the acquisition time it allows the best structure of flip angles and their timings to be found such its averaging extracts the most information about the parameters of interest.

To illustrate the utility of the efficiency concept, figure 1 shows an example simplified ‘fingerprinting’ experiment with only 5 radiofrequency (RF) pulses, each followed by a measurement. When optimized for maximum efficiency in estimating ${T}_{1}$ and ${T}_{2},$ two pulses get set to zero amplitude leaving a 3 pulse, 60°–180°–90°, sequence that basically combines two familiar sequences: a spin-echo and an inversion recovery with optimized inversion and echo times for maximum information. Interestingly, it is more efficient to measure *fewer* signals but allow magnetization to recover, in this case.

Although the results from equations (2) to (3) are general, when computing efficiency with the CRLB we have assumed that each measurement is fully-sampled and acquired using a single channel coil with uniform sensitivity. This allows a quick evaluation of efficiency, crucial for use in optimization, but is not realistic. In practice, data is acquired using multi-channel receiver coils, often with some degree of undersampling, either for parallel imaging or as an inherent part of the method as in MRF. For multi-channel coils, ${\sigma }_{0}$ is still defined as the standard deviation in one readout in one channel, as after pre-whitening all channels should have the same noise level (Pruessmann *et al* 1999). In this case ${M}_{0}$ is the result obtained from optimally combining all channels. Use of multi-channel coils with optimal combination would not affect the efficiency; better intrinsic SNR would improve the $\theta NR$ which is captured by $SN{R}_{max}$ in equation (3). On the other hand, undersampling will affect both ${T}_{acq}$ and ${\sigma }_{\theta },$ impacting the efficiency of the sequence. According to Hu and Peters (2019), the standard deviation $\left({\sigma }_{\theta ,R}\right)$ of $\theta $ in an experiment with an $R$ undersampling factor can be related to the fully-sampled case $\left({\sigma }_{\theta }\right)$ as:

\begin{eqnarray*}\begin{array}{c}\displaystyle \frac{{\sigma }_{{\theta }_{n},R}}{{\sigma }_{{\theta }_{n}}}={d}_{R}\left({\theta }_{n}\right)\sqrt{R},\end{array}\end{eqnarray*}

where ${d}_{R}$ is the so-called ‘dynamics-factor’ that expresses the parameter error amplification due to the ill-conditioning of the parameter estimation (Hu and Peters 2019). The subscript $n$ represents the *n* ^th^ voxel, as ${d}_{R}\,\,$may be spatially varying since it will include effects from coil encoding such as the *g*-factor (Pruessmann *et al* 1999) as well as sampling effects that may arise from use of time-varying non-cartesian *k*-space trajectories. Hence the efficiency of an undersampled experiment ${\eta }_{R}$ can be related back to the fully-sampled experiment efficiency $\eta $ by:

\begin{eqnarray*}\begin{array}{c}{\eta }_{R}\left({\theta }_{n}\right)=\displaystyle \frac{\eta \left({\theta }_{n}\right)}{{d}_{R}\left({\theta }_{n}\right)}.\end{array}\end{eqnarray*}

For a least-squares estimator ${d}_{R}\geqslant 1,$ so can only reduce the efficiency compared to the fully-sampled experiment. In general, ${d}_{R}$ must be estimated by analysis of the full parameter reconstruction pipeline, which will be problem dependant and could become very large for a non-cartesian image reconstruction. In the following subsection we approximate ${d}_{R}$ for the special case of a zero-filled reconstruction.

### Zero-filled reconstruction

The original MRF paper (Ma *et al* 2013) proposed a zero-filled reconstruction that treated undersampling artifacts as noise. In this case for a least-squares estimator the final parameter standard deviations are proportional to the signal standard deviations; thermal noise and ‘aliasing noise’ both contribute noise that have a similar impact on parameter estimation but may differ in relative strength. Assuming ‘aliasing noise’ follows an independent Gaussian distribution $N(0,{\sigma }_{alias}^{2}),$ the image domain signal standard deviation in an undersampled experiment ${\sigma }_{image,R}$ may be written:

\begin{eqnarray*}\begin{array}{c}{\sigma }_{image,R}=\sqrt{{\sigma }_{alias}^{2}+R\cdot {\sigma }_{image}^{2}},\end{array}\end{eqnarray*}

where ${\sigma }_{image}$ is the image domain noise standard deviation in a fully-sampled experiment. Hence, we may write

\begin{eqnarray*}\begin{array}{c}{d}_{R}\approx \displaystyle \frac{1}{\sqrt{R}}\displaystyle \frac{{\sigma }_{image,R}}{{\sigma }_{image}}=\sqrt{\displaystyle \frac{1}{R}\displaystyle \frac{{\sigma }_{alias}^{2}}{{\sigma }_{image}^{2}}\,+1}.\end{array}\end{eqnarray*}

This may further be written in terms of the signal-to-noise-ratio in a ‘fully-encoded’ image ($SN{R}_{image}$) and the signal-to-aliasing ratio ($Sa{R}_{image}$) from an undersampled image without thermal noise as:

\begin{eqnarray*}\begin{array}{c}{d}_{R}\approx \sqrt{\displaystyle \frac{1}{R}\displaystyle \frac{SN{R}_{image}^{2}}{Sa{R}_{image}^{2}}+1}.\end{array}\end{eqnarray*}

It is therefore expected that the dynamics-factor will become larger if the signal-to-noise-ratio of the data improves; although at first this seems non-intuitive it highlights that aliasing effects are proportional to the signal, so a stronger signal leads to a larger contribution. Note that in equations (6)–(8) we have considered a constant signal amplitude and an image domain SNR since aliasing is fundamentally treated in the image domain.

## Method

### Optimized sequence design

In order to make a fair comparison between all analyzed methods, sequence acquisition settings ${\boldsymbol{u}}$ (repeat time, flip angles $\left(\alpha \right)$ etc) were optimized similarly to other works (Gras *et al* 2017, Nataraj *et al* 2017, Teixeira *et al* 2017, Assländer *et al* 2019b, Zhao *et al* 2019), improving ${T}_{1}$ and ${T}_{2}$ efficiencies for a range of tissue parameters ${\boldsymbol{p}}$ represented by the set of parameters $P.$ For clarity, $\theta $ is the set of parameters of interest, while ${\boldsymbol{p}}$ is all parameters that may affect the signal; in general $\eta \left(\theta \right)\equiv \eta \left(\theta ;{\boldsymbol{p}},{\boldsymbol{u}}\right)$ but this dependence is kept implicit for notation simplicity. In this work $\theta =\left\{{T}_{1},{T}_{2}\right\}$ while ${\boldsymbol{p}}$ depends on the types of sequence used—if spoiled sequences are used then ${\boldsymbol{p}}=\left\{{T}_{1},{T}_{2},{M}_{0}\right\},$ but if balanced sequences are used, then off-resonance frequency ${\omega }_{0}$ and phase of the measurement ${\phi }_{0}$ are also relevant, such that ${\boldsymbol{p}}=\left\{{T}_{1},{T}_{2},{M}_{0},{\phi }_{0},{\omega }_{0}\right\}.$

The optimization solved to find the acquisition parameters ${\boldsymbol{u}}$ that maximizes efficiency for each method follows:

\begin{eqnarray*}\begin{array}{c}{{\boldsymbol{u}}}^{opt}=\mathrm{arg}\mathop{\min }\limits_{{\boldsymbol{u}}}\displaystyle \displaystyle \sum _{{\boldsymbol{p}}\in P}{\left(\displaystyle \frac{1}{\eta \left({T}_{1};{\boldsymbol{p}},{\boldsymbol{u}}\right)}\right)}^{2}+{\left(\displaystyle \frac{1}{\eta \left({T}_{2};{\boldsymbol{p}},{\boldsymbol{u}}\right)}\right)}^{2}\\ \,s.t.\,g\left({\boldsymbol{u}}\right)\leqslant 0\,\\ \,f\left({\boldsymbol{u}}\right)=0,\end{array}\end{eqnarray*}

where $g$ and $f$ are method-dependent constraint functions and are detailed in supporting information table S1 (available online at stacks.iop.org/PMB/66/15NT02/mmedia). The set of parameters $P$ consists of ${T}_{1}=781\,{\mathrm{ms}},$ ${T}_{2}=65\,{\mathrm{ms}},$ (corresponding to white matter at 3 T Bojorquez *et al* 2017); ${M}_{0}=1,$ ${\phi }_{0}={0}^{{\mathrm{o}}}$ and ${\omega }_{0}\in \left[-100,100\right]\,{\mathrm{Hz}}$ in steps of 5 Hz. Inclusion of a range of ${\omega }_{0}$ forces methods based on balanced sequences to achieve good efficiencies over a range of frequencies.

The optimization problem was solved using the sequential quadratic program algorithm from Matlab function *fmincon*. Whenever the number of design variables was $\leqslant 400,$ a multi-start strategy was employed consisting of $100$ random initializations, otherwise a single initialization was used consisting of the originally published acquisition settings for the respective method.

### Efficiency comparison

We have studied five steady-state methods: DESPOT (Deoni *et al* 2003) and a variant called joint system relaxometry (JSR) (Teixeira *et al* 2017) (analyzed together due to their similarity), PLANET (Shcherbakova *et al* 2018), DESS (Welsch *et al* 2009) and triple echo steady-state (TESS) (Heule *et al* 2014); and MRF sequences with gradient spoiled (Jiang *et al* 2015) or balanced (Ma *et al* 2013, Assländer *et al* 2016) readouts. MRF sequences started from thermal equilibrium or were in a DE mode (Ma *et al* 2018; Assländer *et al* 2019b), in which a pulse train of fixed length is cycled such the final magnetization is equal to the initial magnetization. For each method optimized acquisition settings ${{\boldsymbol{u}}}^{opt}$ were determined using equation (9) with efficiency calculated using the CRLB in equation (3) assuming fully-sampled measurements. This was repeated for several acquisitions with different numbers of measurements (table 1). The signal model, optimization constraints and acquisition settings for each method are in supporting information table S1.

**Table 1.** Number of measurements of the several acquisitions for which the ${T}_{1}$ and ${T}_{2}$ efficiencies of every method were optimized. $N$ is the number of measurements in the transient method (length of the fingerprint). For the transient methods (in orange), fingerprints with less than 400 measurements (in gray) were not considered for further analysis as these could be incompatible with spatial encoding.

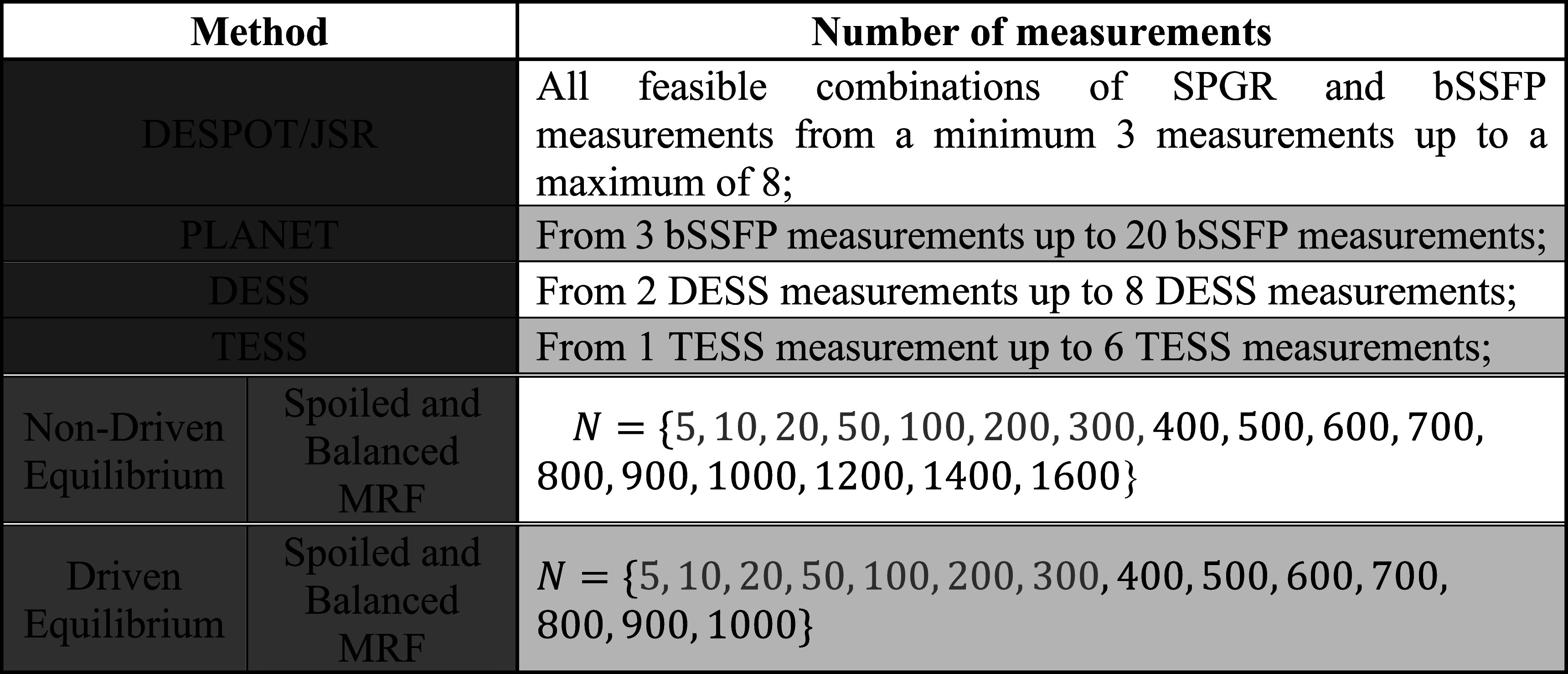

For each method the acquisition with the lowest cost function (highest efficiency) was evaluated for a wider range of ${T}_{1}\in \left[600,1200\right]\,{\mathrm{ms}}$ in steps of 40 ms and ${T}_{2}\in [40,100]\,{\mathrm{ms}}$ in steps of 4 ms. We found that MRF sequences with very small numbers of pulses can achieve very high efficiencies, particularly if starting from thermal equilibrium. However, in practice such short pulse trains make only a limited number of measurements, so cannot support spatial encoding. Therefore, MRF acquisitions with less than 400 excitations were not evaluated over the extended parameter range (values shown in gray in table 1).

For the main cross comparisons between methods, all efficiencies were calculated assuming fully-sampled measurements. This makes a concise and transparent presentation and is a reasonable approach for steady-state methods. However, MRF methods are most often acquired with a considerable degree of undersampling, and in these cases the obtained efficiency would also depend on the dynamics-factor ${d}_{R}$ according to equation (5). To address this we estimated ${d}_{R}$ for the sub-optimal zero-filled reconstruction using equation (8). Both random and spiral (Glover 1999, Pipe and Zwart 2013, Wundrak *et al* 2015) undersampling were explored for the Shepp–Logan phantom and Monte-Carlo simulations (100 000 trials each with independent Gaussian additive noise) were performed to estimate the standard deviation of the undersampled data ${\sigma }_{image,R}.$ Several undersampling factors $R$ and different SNR levels were used to estimate ${d}_{R}.$

### Validation experiments

To experimentally validate the efficiency metric the standard deviation of $\theta $ needs to be determined, requiring multiple estimates obtained from different datasets. To stay within an acceptable acquisition time, we focused on validation of efficiency prediction for estimation of a single parameter—${T}_{1}$ using DESPOT1 (Christensen *et al* 1974). One healthy volunteer (male, age 25) was scanned on 3 T Achieva MRI systems (Best, Netherland) using a 32-channel head coil. Brain images at a resolution $1\times 1\times 3\,{\mathrm{mm}}$ were obtained using 3D Cartesian encoding of a transverse slab with 7 slices, such that the middle slice could be analyzed free of slice profile effects; no parallel imaging acceleration was used. The acquisition consisted of $10$ repeats of $6$ SPGR sequences with $\alpha =\left\{{5}^{\circ },{8}^{\circ },{10}^{\circ },{13}^{\circ },{15}^{\circ },{18}^{\circ }\right\}$ and ${\mathrm{TR}}=20\,{\mathrm{ms}},$ yielding a maximum root mean squared RF field of 0.46 *μ*T for $\alpha ={18}^{\circ }$ to minimize bias from magnetization transfer (MT) effects (Ou and Gochberg 2008, Teixeira *et al* 2019a). To compare multiple examples with different efficiencies, all combinations of at least 3 SPGR were considered. Additionally, a transmit field (${B}_{1}^{+}$) map was acquired using actual flip angle imaging method (Yarnykh 2007) with an isotropic resolution of $5\,{\mathrm{mm}}.$

All numerical simulations and analyses were performed in a workstation with 64 GB of RAM and with an Intel Xeon E5-2687 W 0 @ 3.10 GHz, using MATLAB R2017b (The MathWorks, Natick, MA, USA) with some functions implemented in C++/MEX using the Eigen linear algebra library (Guënnebaud and Jacob 2010).

## Results

### Efficiency comparison

**Figure 2.**
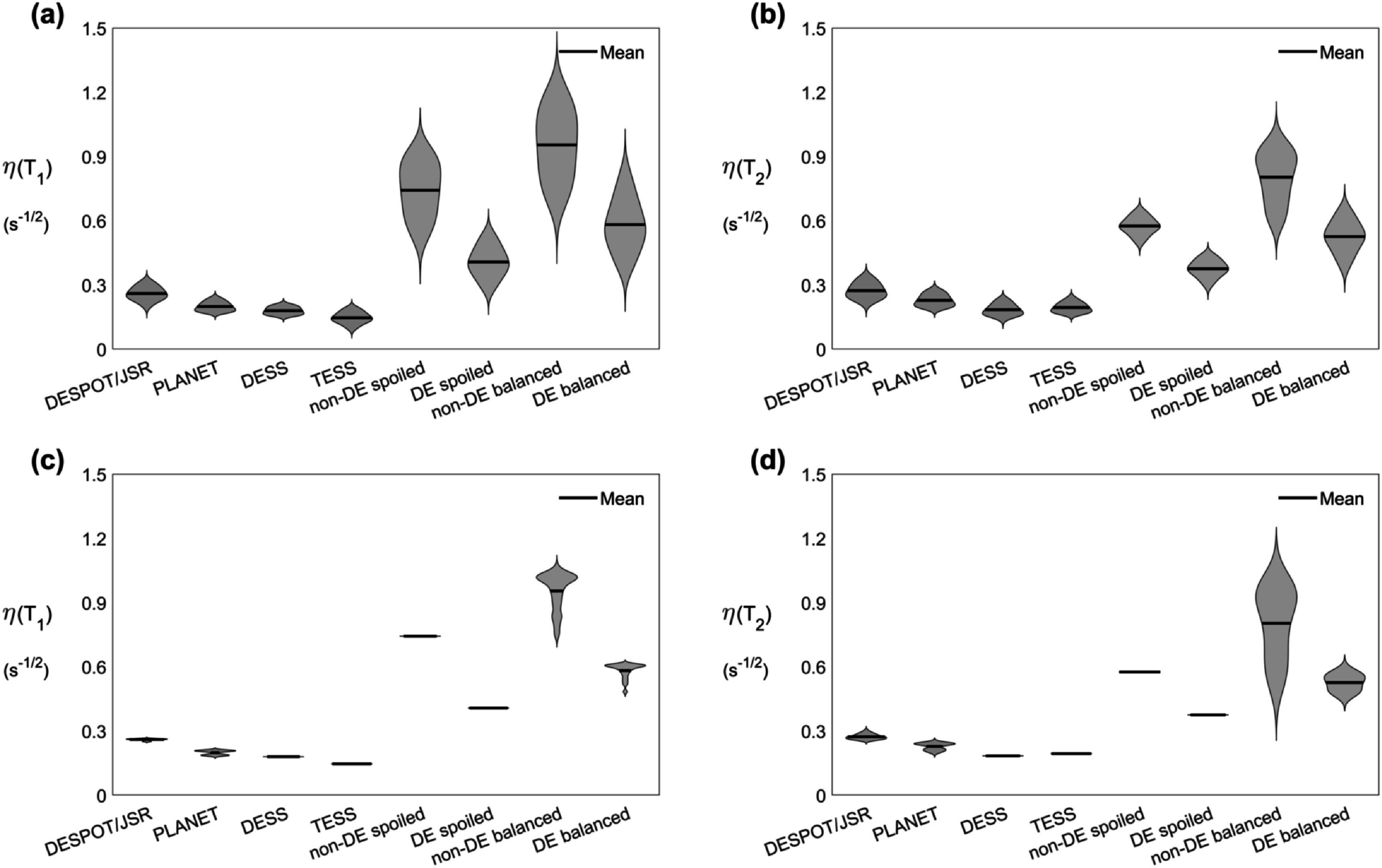
Efficiency comparison for steady-state (blue) and transient (orange) methods as described in subsection ‘efficiency comparison’; in each case the results shown are for the most efficient acquisition of each method (table 1). (a), (b) ${T}_{1},{T}_{2}$ efficiency averaged over all off-resonance values; spread corresponds to variability over $\left\{{T}_{1},\,{T}_{2}\right\}.$ (c), (d) ${T}_{1},{T}_{2}$ efficiency averaged over all {${T}_{1},$ ${T}_{2}$}; spread corresponds to variability over off-resonance frequencies. As may be expected the balanced sequences show greater sensitivity to off-resonance.

**Figure 3.**
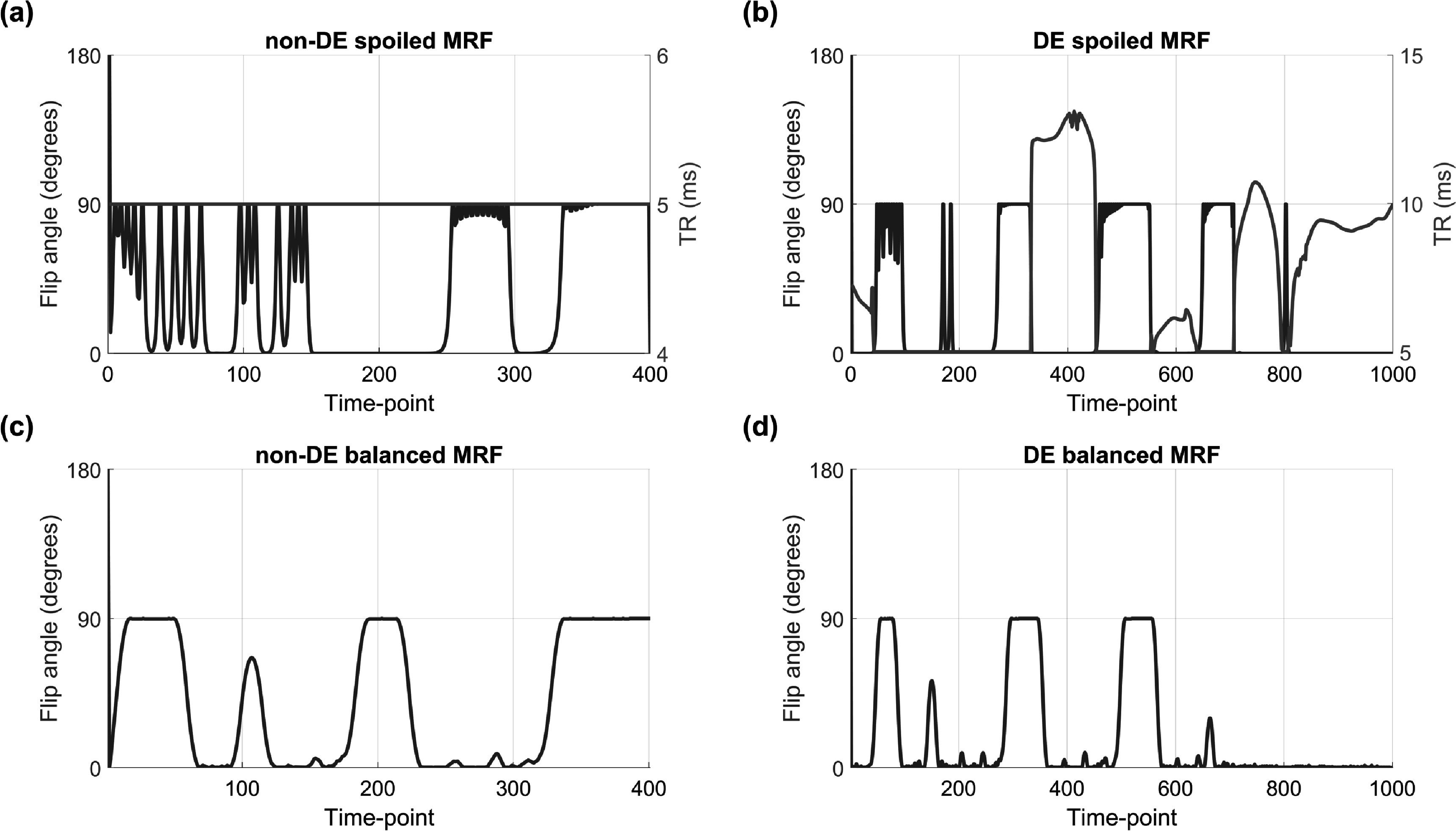
Optimized settings for each of the transient methods used for cross comparisons. In spoiled MRF (a) and (b) both flip angle and TR were optimized, whilst for balanced MRF (c) and (d) only flip angle was optimized (TR fixed at 5 ms). For those starting from thermal equilibrium (a) and (c) the shortest feasible sequence was the most efficient (*N* = 400; see table 1), whereas the opposite was verified for driven equilibrium (b) and (d) sequences (*N* = 1000; see table 1).

Figure 2 shows a comparison of optimized efficiencies for steady-state and transient methods assuming fully-sampled measurements in all cases, whilst the optimized acquisition settings are in supporting information table S2 and figure 3. Figures 2(a) and (b) show the distribution of efficiency values over different ${T}_{1}$ and ${T}_{2}$ values and averaged over off-resonance, while figures 2(c) and (d) show spread over different off-resonance values averaged over ${T}_{1}$ and ${T}_{2}$ values. Consistently we see that the steady-state methods are less efficient than their transient counterparts; DESPOT/JSR is the most efficient steady-state method while balanced MRF starting from thermal equilibrium is the most efficient transient method. In general the best transient method is approximately 3–3.5 times more efficient than the best steady-state method. The transient methods have an apparently greater spread in efficiency as a function of ${T}_{1}$ and ${T}_{2}.$ Only the methods that include balanced readouts are sensitive to off-resonance, and of these the transient methods seem more sensitive than the steady-state ones. Nevertheless, methods using balanced readouts are more efficient than those using spoiled readouts despite having to estimate two additional nuisance parameters. The optimized acquisition settings for the best transient sequences in figure 3 also reveal recovery periods of zero flip angle in lieu of performing more measurements. Supporting information figure S1 compares efficiency of different optimized MRF trains with different numbers of measurements.

**Figure 4.**
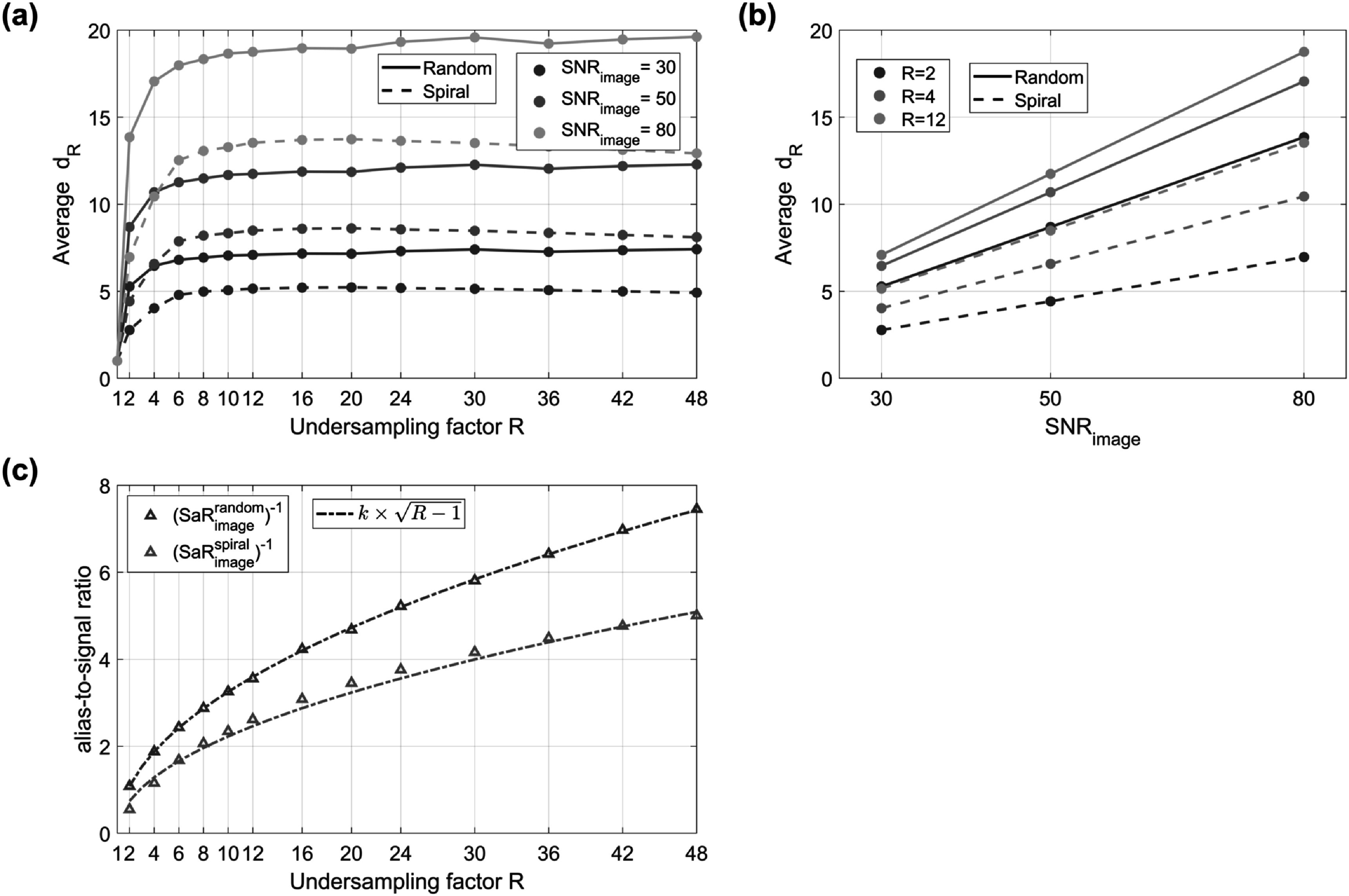
Analysis of the effect of undersampling for random and spiral sampling. (a) Average ${d}_{R}$ in the non-zero locations of the Shepp–Logan phantom as a function of the undersampling factor *R* and (b) as a function of the SNR in the image domain. (c) Aliasing-to-signal ratio as a function of the undersampling factor *R* and its empirical fit to the expression $k\times \sqrt{R-1}.$

Figure 4 shows the results of the Monte-Carlo investigation of the dynamics-factor ${d}_{R}$ for zero-filled reconstructions computed with random and spiral sampling. For this special case, ${d}_{R}$ increases quickly for lower undersampling factors *R*, but then plateaus at higher *R*; the level it reaches is directly proportional to $SN{R}_{image}$ (figure 4(b)), with spiral sampling achieving lower ${d}_{R}$ values than random sampling. Figure 4(c) plots the ‘aliasing-to-signal ratio’ as a function of *R* for a scenario with zero thermal noise; empirical fits to data show that to a good approximation this is proportional to $\sqrt{R-1}\,\,$for both sampling schemes used. The full ${d}_{R}$ maps are in supporting information figure S2.

### *In vivo* validation

**Figure 5.**
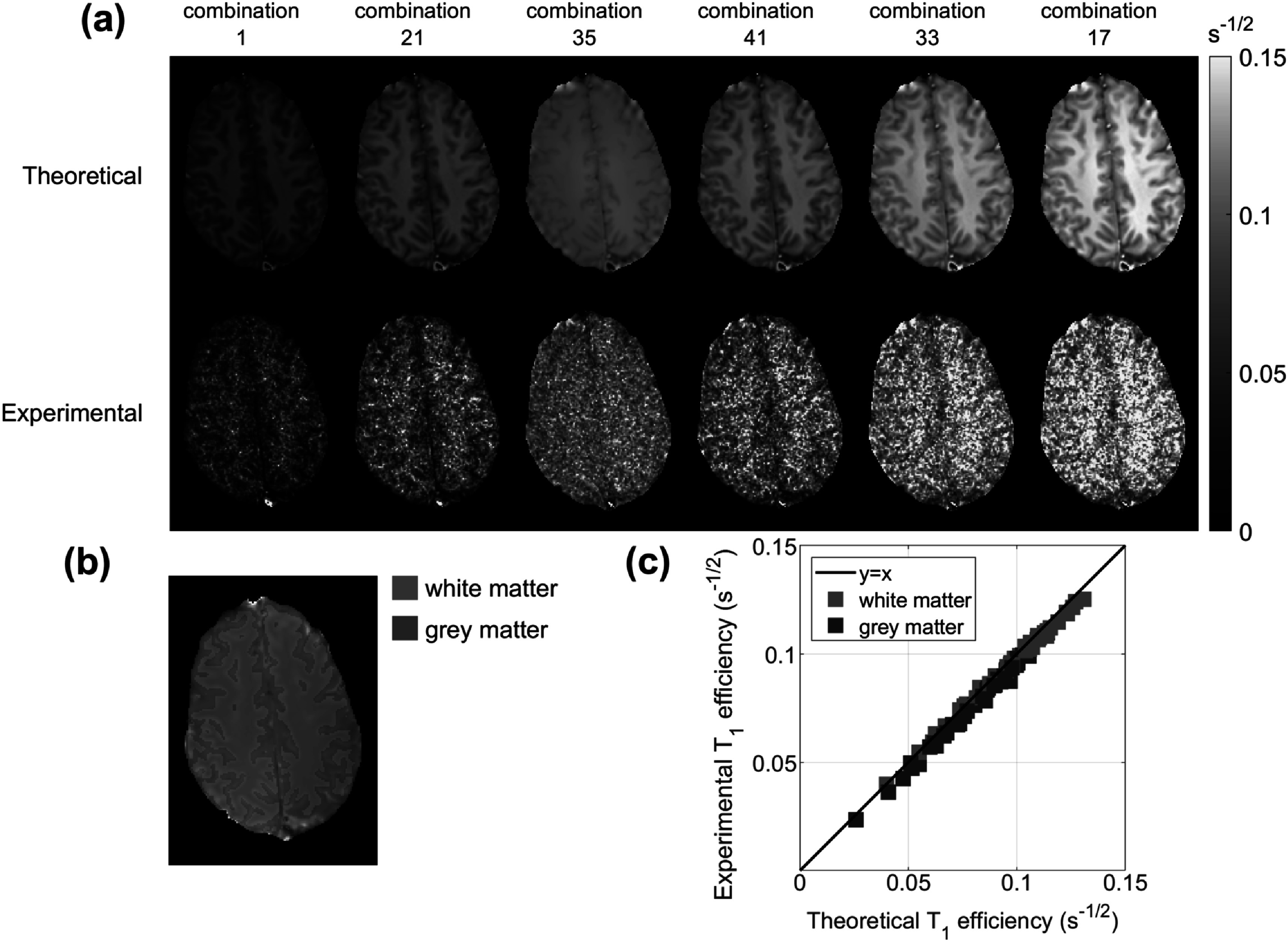
*In vivo* validation results for the ${T}_{1}$ efficiency of DESPOT1. (a) Theoretical and experimental ${T}_{1}$ efficiency maps obtained for some combinations of the acquired SPGRs. (b) White and gray matter masks obtained using FSL FAST (Zhang *et al* 2001). (c) Average experimental ${T}_{1}$ efficiency inside the gray and white matter masks plotted against the respective theoretical efficiency for each combination of SPGRs. A table with all SPGR combinations is provided in supporting table S3 and all maps are in supporting information figure S3. The correction for incomplete spoiling in SPGR proposed by Baudrexel *et al* (2018) was implemented. Brain extraction was performed using FSL BET (Smith 2002) and all images were registered using MIRTK (Schuh *et al* 2013). Gold standard values for ${T}_{1}$ and ${M}_{0}$ were estimated from fitting to the average of all repetitions and the noise standard deviation ${\sigma }_{0}$ was estimated from the image domain (supporting information figure S4).

Figure 5 depicts comparisons of ${T}_{1}$ efficiency for DESPOT1 estimation *in vivo* for several subsets of the total dataset. Figure 5(a) shows efficiency maps for a selection of combinations (the full set of maps is in supporting information figure S3). Figure 5(c) shows the average of the experimental ${T}_{1}$ efficiency inside the white and gray matter regions (figure 5(b)) plotted against the average of the theoretical efficiency in the same regions for each combination tested. The goodness-of-fit for this comparison was calculated to be ${{R}}^{2}=0.9688.$

## Discussion

This work presented a comparison of qMRI methods for simultaneous ${T}_{1}$ and ${T}_{2}$ estimation based on an efficiency metric, $\eta ,$ that quantifies the information encoded about the parameters per square root of acquisition time. The efficiency metric was validated *in vivo* using the DESPOT1 method (figure 5). The results showed an excellent agreement between experimental and theoretical ${T}_{1}$ efficiency (${{R}}^{2}=0.9688$) when averaged over white/gray matter masks.

The metric $\eta $ considers intrinsic efficiency related to the dynamics of the spin system only. Therefore, it considers what mainly distinguishes one method from another: the intrinsic way magnetization is manipulated to achieve greater or lesser sensitivity to the tissue parameters of interest. Nevertheless, according to equation (2) there are two other factors that affect the final parameter-to-noise-ratio, and these do need to be considered in the bigger picture. One factor is the amount of data acquired, as efficiency is normalized by $\sqrt{{T}_{acq}}.$ Thus, the SNR advantage yielded by 3D encoding compared to 2D encoding should be accounted for when comparing the expected parameter-to-noise-ratio. The other is the intrinsic SNR of the experiment, specified by $SN{R}_{max}.$ While increasing the repetition time might appear to reduce efficiency, one could for example make use of that time to reduce the receiver bandwidth, thus increasing $SN{R}_{max}.$ Moreover, different *k*-space sampling strategies could be employed to make more efficient use of that time, like spiral sampling (Jiang *et al* 2015) or even EPI (Rieger *et al* 2017, 2018). However, there are pragmatic limits to which such trade-off can be explored due to ${T}_{2}^{* }$ decay and increased sensitivity to water-fat shift, off-resonance, among others. In the end, intrinsic SNR, amount of data acquired, and efficiency have to weighed together to determine the best method for a specific application.

A Cramér–Rao approach was adopted for determining the uncertainty of parameter estimation, providing a secure mathematical basis for interpretation of results with a clear domain of applicability based on unbiased estimators (Sengupta and Kay 1995). This connects to a key assumption in relaxometry—the biophysical model. In this work we assumed that the magnetization dynamics are well represented by the Bloch equations and that each voxel contains only one tissue type. In practice this ignores factors such as MT (Ou and Gochberg 2008, Teixeira *et al* 2019a, 2019b) and diffusion (Kobayashi and Terada 2018) that can produce systematic differences between the model and data, biasing parameter estimation, and to which different methods have different sensitivities.

Figure 2 suggests that after optimization, the transient methods are generally more efficient than the steady-state methods for fully-sampled experiments. From the latter, DESPOT/JSR has the highest optimized efficiency. This approach combines SPGR and balanced SSFP sequences, giving it a large degree of flexibility. We found that the most efficient acquisitions consisted of mainly bSSFP sequences but having at least one SPGR enhances efficiency by decorrelating ${T}_{1}$ and ${T}_{2}$ information. The PLANET method uses only bSSFP sequences, which results in slightly lower efficiency as it constitutes a constrained case of JSR that excludes SPGR measurements and forces a single flip angle and TR. Optimizing bSSFP sequences to maximize $\eta $ leads to use of multiple different flip angles. Because TESS and DESS obtain multiple echoes per TR period, they are often thought of as efficient, however our results indicate this may not be the case. Although they measure multiple echo pathways, the information in these echoes is correlated since they share the same flip angle and TR, and the higher order echoes often have lower signal amplitudes; it could be more efficient to obtain more diverse data instead.

The transient (MRF) sequences are divided into those in DE and those that start from thermal equilibrium, and between either gradient spoiled or balanced readouts. The results in figure 2 compared only the most efficient transient acquisition with at least 400 measurements (figure 3). Supporting information figure S1 expands this with efficiencies for different numbers of measurements. It was seen that very high efficiencies can be achieved for short MRF pulse trains that start from thermal equilibrium; these may not be viable for performing the spatial encoding required for 3D but could potentially be used for 2D encoding. Methods that start from thermal equilibrium (i.e. the ‘non-DE’ sequences) are ‘privileged’ by assuming sufficient time has elapsed following previous acquisitions to allow full recovery. If this recovery time is also included in the efficiency calculation these methods lose their high efficiency. In the case of longer acquisitions, both MRF sequences in DE and starting from thermal equilibrium converged to a similar efficiency, which is still higher than steady-state methods.

Overall the best transient method was found to be 3–3.5 times more efficient than the best steady-state method considering fully-sampled measurements. It is important to note that this apparently superior efficiency comes from considering spin dynamics alone, i.e. information encoded into the data, but excludes spatial sampling and reconstruction. Our analysis assumed that reconstruction faithfully represents the state of the system at each timepoint. In practice undersampling of the data, which is particularly a feature of transient methods (Ma *et al* 2013), reduces efficiency according to the dynamics-factor ${d}_{R}.$ This factor depends on the reconstruction method. Many such methods are available—an exhaustive review of these is beyond the scope of this study. The quoted efficiencies excluding ${d}_{R}$ could thus be considered best-case scenarios. At the other extreme, a zero-filled reconstruction (initially proposed for MRF Ma *et al* 2013) is a straightforward entry-level approach that represents a reasonable worst case for ${d}_{R}.$ Figure 4 shows that for higher undersampling factors *R*, ${d}_{R}$ is independent of *R* but scales linearly with the SNR of the data. To understand these relationships, figure 4(c) shows empirically that the ‘alias-to-signal’ ratio scales as $\sqrt{R-1}.$ Substituting this into equation (8) we find:

\begin{eqnarray*}{d}_{R}\approx \sqrt{\displaystyle \frac{k\left(R-1\right)}{R}SN{R}_{image}^{2}+1},\end{eqnarray*}

where $k$ is a scaling constant, which yields ${d}_{R}\propto SN{R}_{image}$ for large *R* and large SNR. Though at first unintuitive this should be expected since high SNR implies that the dominant source of ‘noise’ in the reconstruction comes from aliasing. This implies that actually MRF using a simple zero-filled reconstruction will be less efficient than steady-state imaging in many circumstances.

Of course, it is important to reiterate that zero filling is a simple but sub-optimal approach to MRF reconstruction, and many more sophisticated and superior methods do exist. Current reconstructions using machine learning (Cohen *et al* 2018, Hamilton and Seiberlich 2020, Hermann *et al* 2021) and/or some prior knowledge or form of regularization (Pierre *et al* 2016, Assländer *et al* 2018a, Zhao *et al* 2018) should be expected to reduce ${d}_{R}$ since they attempt to resolve the aliased signal rather than treat it as noise (Stolk and Sbrizzi 2019). Hence the ${d}_{R}$ values computed here might be considered as upper bounds, yet it is still to be shown how far ${d}_{R}$ can be reduced by more modern methods while still maintaining accuracy. A further consideration is that more complex reconstructions employing regularization or prior knowledge may introduce bias, which is beyond the scope of the proposed efficiency metric that considers only precision assuming unbiased estimation.

Our comparison analyzed error amplification from thermal noise considering gradient-echo based methods for brain imaging that simultaneously estimate ${T}_{1}$ and ${T}_{2}.$ For other applications (e.g. cardiac or abdominal imaging) different considerations (e.g. physiological noise, SAR, or motion) need to be taken into account. These can be incorporated by changing the model and/or constraining the design space, which might change the comparison landscape obtained here, making particular methods unviable and could favor methods that would otherwise seem inefficient.

## Conclusion

This work presents a comparison of several qMRI methods based on an efficiency metric that is the ratio of the best-case parameter-to-noise-ratio to the maximum achievable SNR, normalized to the square root of the acquisition time. This allows the performance of different qMRI methods to be quantified and optimized, and has been used here to compare a range of well-established methods that simultaneously estimate ${T}_{1}$ and ${T}_{2}$. We found that methods based on balanced readouts outperformed methods based on spoiled readouts, and that transient qMRI sequences can be 3–3.5 times more efficient than steady-state alternatives for both ${T}_{1}$ and ${T}_{2}$ mapping in fully-sampled experiments. However, transient sequences which need to capture rapidly fluctuating signals often deploy highly undersampled acquisition strategies. The manner with which this undersampling is resolved can reduce the overall efficiency. For example, MRF using zero-filled reconstruction that treats aliasing artifacts as noise (Ma *et al* 2013), could easily drop efficiency by a factor of $5,$ thereby negating the gains offered by a transient acquisition. More advanced reconstructions will be important to realize the potential gains offered by MRF.

The concept of an efficiency metric, such as the one proposed in this paper, can capture all aspects of both acquisition and reconstruction providing a means to assess relative performance and so can help in selection of optimal qMRI methods.

## Ethical statement

Healthy volunteer gave written informed consent according to local ethics requirements (Guy’s Research Ethics Committee, ID 01/11/12).
